# Secreted mitochondrial aspartyl‐tRNA synthetase (DARS2) regulates TNFα signaling

**DOI:** 10.14814/phy2.70627

**Published:** 2025-11-10

**Authors:** Benjamin S. Johnson, Alex Cornwell, Daniela Farkas, Ilknur Yurtsever, Jessica A. Joseph, Arun Pradhan, Laszlo Farkas, James D. Londino, Joseph S. Bednash, Rama K. Mallampalli

**Affiliations:** ^1^ Department of Internal Medicine, Division of Pulmonary, Critical Care, and Sleep Medicine The Ohio State University Columbus Ohio USA

**Keywords:** cytokine, DARS2, lung epithelia, mitochondria

## Abstract

Aminoacyl‐tRNA Synthetases (aaRS) are important regulators of cytokine signaling. Multiple cytoplasmic aaRS family members have been observed to be secreted in response to various stimuli to modulate downstream responses, however, agonist‐induced cellular release of aaRS from mitochondria has not been described. In particular, TNFα is a potent mediator of aaRS release. BEAS‐2B cells were utilized to study the release of mitochondrial Aspartyl‐tRNA Synthetase (DARS2) in response to various cytokines. The role of DARS2 in paracrine signaling was evaluated using adoptive media transfer from BEAS‐2B to recipient THP1 cells. To identify pathways governing DARS2 secretion, blocking antibodies chemical inhibitors and siRNA technology was employed. Herein, we describe DARS2 as the first mitochondrial aaRS released in response to TNFα from airway epithelia. Once secreted, DARS2 binds to macrophages, is internalized, thereby inducing an M1‐like phenotype in recipient macrophages. DARS2 release from airway epithelia is in part, TNFα‐receptor 1 dependent, and requires the endosomal sorting complex required for extracellular transport.

## 1 INTRODUCTION

Mitochondrial Aspartyl‐tRNA‐Synthetase (DARS2) is a mitochondrial tRNA ligase responsible for the conjugation of aspartic acid to its respective tRNA (Isohanni et al., 2010). Additionally, DARS2 is known to mediate neuroinflammation, wound healing, and support the host response to bacterial infections through its actions as a secreted protein (Aradjanski et al., 2017; Johnson et al., 2024; Willenborg et al., 2021). As with other tRNA‐synthetases, DARS2 is secreted from multiple cell types during infection to potentiate the immune response. The mechanism by which DARS2 is secreted remains unknown. Further exploration of this mechanism may identify novel therapeutic targets for immunomodulatory drugs.

An abundance of research shows tRNA synthetases are secreted by a variety of cells to support the immune response (Ahn et al., 2021; Gupta et al., 2023). Many of these are released in response to a variety of bacterial and viral stimuli. Our laboratory was the first to demonstrate a mitochondrial family member, DARS2, acting in this manner to confer innate immune responses in the lung. In addition to acting as immune mediators during infection, tRNA synthetases are important mediators of cytokine secretion both intracellularly and via secretion. Tumor necrosis factor‐alpha (TNFα), in particular, is a potent stimulator of tRNA synthetase secretion. For example, the enzyme Lysyl‐tRNA synthetase (KARS) is released from epithelia in response to TNFα to potentiate further TNFα signaling and immune cell development (Lim et al., 2018; Park et al., 2005). Additionally, both cysteinyl‐ (CARS) and threonyl‐tRNA (TARS) synthetases are secreted in response to TNFα stimulation of endothelia and microglia, respectively (Jung et al., 2020; Qi et al., 2024). Secreted CARS acts in a similar positive feedback manner to KARS by enhancing further TNFα secretion (Qi et al., 2024). While DARS2 has been demonstrated to be released during infection and acts to stimulate cytokine secretion and immune cell chemotaxis, its role in response to cytokine signaling remains unknown (Johnson et al., 2024). Further the mechanism of tRNA synthetase secretion, including DARS2, remains largely unknown.

## 2 MATERIALS AND METHODS

### 2.1 Cell culture and reagents

BEAS‐2B (Human Bronchial Epithelial cells, ATCC, catalog no. CRL‐35588), THP‐1 (Tohoku Hospital Pediatrics‐1, an acute myeloid leukemia line used as a model of monocytes, ATCC, catalog no. TIB‐202), primary HSAEC (human small airway epithelial cells, ATCC, catalog no. PCS‐301‐010), and primary HBEC (human bronchial epithelial cells, ATCC, catalog no. PCS‐300‐010) were used for this study. BEAS‐2B cells were cultured in HITES media, a DMEM/F12 media (Gibco, catalog no. 21331020) supplemented with 10% FBS (GeminiBio, catalog no. 100‐106‐500), insulin‐transferrin, and selenium (all from Gibco, catalog no. 41400045), hydrocortisone (Sigma‐Aldrich, catalog no. H0135), β‐estradiol (Sigma‐Aldrich, catalog no. E2758), HEPES (Gibco, catalog no. 15630080), L‐glutamine (Gibco, catalog no. 25030081), and penicillin/streptomycin (Gibco, catalog no. 15140122). THP‐1 cells were maintained in complete 10% FBS/RPMI (Sigma‐Aldrich, catalog no. R8758) media and differentiated with 20 ng/mL PMA (phorbol 12‐myristate 13‐acetate, Sigma‐Aldrich, catalog no. P1585) for 72 h. Primary HSAEC and HBEC were cultured according to ATCC in Airway Epithelial Cell Basal Media (ATCC, catalog no. PCS‐300‐030) supplemented with components within the Bronchial Epithelial Cell Growth Kit (ATCC, catalog no. PCS‐300‐040) and used in low passages. HAM (human alveolar macrophages) were isolated from failed donor lungs and obtained from the Comprehensive Transplant Center (CTC) human tissue biorepository at OSU and cultured in complete 10% FBS/RMPI (Sigma‐Aldrich, catalog no. R8758) media. Cells were treated with variable concentrations of TNFα (125‐1000 ng/mL, PeproTech, catalog no. 300‐01A), IL‐1β (500 ng/mL, PeproTech, catalog no. 200‐01B), IFNγ (200‐500 ng/mL, PeproTech, catalog no. 300‐02) and IFNβ (200 ng/mL, PeproTech, catalog no. 300‐02BC).

### 2.2 Gene expression

RNA was extracted from cells using the RNeasy Plus Mini Kit (Qiagen, catalog no. 74134) according to the manufacturer's protocol. High‐Capacity RNA‐to‐cDNA™ (Applied Biosystems, catalog no. 4387406) was used for reverse transcription reactions to produce cDNA. Expression levels were quantified by RT‐qPCR on a CFX96 Real‐Time System (Bio‐Rad) using the iTaq universal SYBR Green supermix (Bio‐Rad, catalog no. 172524). Primers used are shown in Table S1.

### 2.3 Plasmid and siRNA transfections

For transient overexpression, plasmid(s) were mixed with X‐tremeGENE HP (Roche, catalog no. 6366244001) at a ratio of 1 μg:2 μL in 300 μL OptiMEM (Gibco, catalog no. 31985062) per reaction. pcDNA 3.1 DARS2‐FLAG tagged and pcDNA 3.1 Empty vector (EV) FLAG tagged plasmids were used as described (Johnson et al., 2024). For siRNA studies, cells were transfected with control and gene of interest siRNA [20 nM] in a reaction mixture of 1:50 GenMute: 1× GenMute Buffer (SignaGen Laboratories, catalog no. SL100568). Reaction volume was dependent on well size. RNAi sequences are listed in Table S2.

### 2.4 Cell functional studies

Mitochondria OCR and dynamics were assayed using a Seahorse XFe96 analyzer (Agilent). A Seahorse XF Cell MitoStress Kit (Agilent, catalog no. 103015‐100) was used to conduct these experiments and was used according to protocols provided by the company. Cytokines were assayed via multiplex ELISA (Meso Scale Discovery, catalog no. K15345D‐1) according to the manufacturer's protocol or individual ELISAs for IL‐6 (Invitrogen, catalog no. 88706686) and TNF‐α (Invitrogen, catalog no. 88734686). Cell death was measured using CyQUANT LDH Cytotoxicity Assay (Invitrogen, catalog no. C20300) according to the manufacturer's instructions. For studies with THP1‐derived macrophages, cells were seeded at the desired density and treated with PMA (20 ng/mL, Sigma‐Aldrich, catalog no. P1585) for a minimum of 72 h to induce differentiation into macrophages.

### 2.5 Media transfer experiments

THP‐1 cells were differentiated to macrophages using 20 ng/mL PMA (Phorbol 12‐myristate 13‐acetate) at a density of 2 × 10^5^ cells/cm^2^ for 72 h. BEAS‐2B cells were transfected as described above with pcDNA DARS2 3× FLAG and pcDNA EV 3× FLAG (control vector). 24 h after transfection, the cell culture media were changed to wash out remaining plasmid. Then, 48 h after transfection, concentrated cell culture media from BEAS‐2B were transferred to recipient THP‐1 macrophages for 6 or 24 h. Before transferring media to THP‐1 macrophages, concentrated media were centrifuged for 3 min at 1000 × g. For immunofluorescence staining and confocal microscopy, endosomal and proteasomal inhibitors were added during media transfer to THP‐1 macrophages (MG132 [100 μM, UBPBio, catalog no. F1101], bafilomycin A1 [200 nM, Sigma‐Aldrich, catalog no. B1793], and the late endosome trafficking inhibitor [EGA20 μM, Sigma‐Aldrich, catalog no. SML1006]). For gene expression of pro‐inflammatory cytokines in recipient THP‐1 macrophages, cell culture media from BEAS‐2B were concentrated with 10 kD Amicon Ultra‐0.5 centrifugal filter devices (Millipore, catalog no. UFC501024).

### 2.6 Immunoblotting

Cells were lysed in Protein Lysis Buffer A: PBS with 0.2% SDS (RPI, catalog no. L22040), 0.05% 100X‐Triton (Sigma‐Aldrich, catalog no. 93443), and 1 Pierce Protease Inhibitor Tablets/10 mL (Thermo Scientific, catalog no. A32963) and then sonicated for 20 s at 25% on a VibraCell Sonicator (Sonics). Lowry Assay (Bio‐Rad, catalog no. 5000111) was used to measure protein concentration. Laemmli buffer (Bio‐Rad, catalog no. 1610747) was used to dilute samples, and precast gradient mini‐ or midi‐gels used for SDS‐PAGE were then transferred to nitrocellulose membranes via Trans‐Blot Turbo Transfer apparatus. Membranes were scanned using a Chemidoc MP imager (Bio‐Rad). All immunoblots were used for densitometry analysis utilized software from Bio‐Rad, ImageJ (NIH), or Image Lab (Bio‐Rad). Antibodies used in these studies were anti‐DARS2 (ProteinTech, catalog no. 13807‐1‐AP, 1:2000), anti‐IARS2 (ProteinTech, catalog no. 17170‐1‐AP, 1:2000), anti‐β‐actin (Sigma‐Aldrich, catalog no. A5441, 1:5000), anti‐FLAG (Millipore, catalog no. F1804, 1:2000), and p‐p65 (Cell Signaling, catalog no. 3033, 1:2000), p65 (Cell Signaling Technologies, catalog no. 8042, 1:2000), and anti‐ubiquitin (Cell Signaling, catalog no. 3936). Secondary antibodies, goat anti‐rabbit IgG (H + L)‐HRP conjugate (Bio‐Rad, catalog no. 1706515) and goat anti‐mouse IgG (H + L)‐HRP conjugate (Bio‐Rad, catalog no. 1706516), were used for immunoblotting.

### 2.7 Supernatant collection

All studies examining supernatant protein levels were conducted using FBS‐free culture media. Protein was concentrated from supernatant using Amicon Ultra‐0.5 Centrifugal Filter Devices (Millipore, catalog no. UFC501024) according to company protocol. Concentrated supernatants were diluted in Protein Lysis buffer A and then processed as above.

### 2.8 Immunoprecipitation (IP)

Cells were incubated with the proteasome inhibitor MG132 (100 μM, UBPBio, catalog no. F1101) and lysosome inhibitor bafilomycin A1 (200 nM, Sigma‐Aldrich, catalog no. B1793) prior to lysis. Protein lysis buffer A, containing deubiquitinase inhibitors 1,10‐phenanthroline (Sigma‐Aldrich, catalog no. 131377), PR‐619 (Sigma‐Aldrich, catalog no. 662141), and *N*‐ethylmaleimide (Thermo Fischer, catalog no. 23030), was used for cell lysis. Lysates were processed as described above. Cells were then incubated while rotating at RT for 30 min with anti‐FlagM2 Magnetic Beads (Millipore, catalog no. F1804), washed thrice with IP wash buffer (0.2% Triton‐X100 (Sigma‐Aldrich, catalog no. 93443, in PBS)), and then eluted in 2× Laemmli (Bio‐Rad, catalog no. 1610737) via boiling.

### 2.9 Administration of recombinant proteins

Recombinant human DARS2 (OriGene Technologies, Inc., catalog no. TP307360) was packaged in Lipofectamine 3000 (Invitrogen, catalog no. L3000001) according to the manufacturer's instructions. These reactions were suspended in 25 μL optiMEM (Gibco, catalog no. 31985062), incubated at room temp for 15 min, and added to cell culture in a dropwise fashion.

### 2.10 Receptor blocking experiments

For antigen receptor blocking experiments, BEAS‐2B cells were incubated with anti‐TNFR1 antibody (Sino Biologicals, catalog no. 10872‐R111), anti‐TNFR2 antibody (Sino Biologicals, catalog no. 10417‐R00N6), or normal rabbit control IgG antibody (Sino Biologicals, catalog no. CR1) at various concentrations (10–500 ng/mL) for 1 h. After incubation, the antibody‐containing medium was removed, and the cells were washed with PBS to eliminate unbound antibodies. The cells were then cultured in serum‐free medium supplemented with or without 500 ng/mL TNFα (PeproTech, catalog no. 300‐01A) for 6 h. Following treatment, cell culture supernatants were collected for assessment of DARS2 secretion, and cell lysates were prepared for immunoblotting.

### 2.11 DARS2 secretion studies

BEAS‐2B cells were cultured in serum‐free medium supplemented and pre‐treated with 0.5 μM MLN7243 (Cayman Chemical, catalog no. 30108) for 30 min. After incubation, the cells were treated with 500 ng/mL TNFα or vehicle (DMSO) for 4 h. Following treatment, cell culture supernatants were collected for assessment of DARS2 secretion, and cell lysates were prepared for immunoblotting.

### 2.12 Immunofluorescence staining/confocal microscopy

THP‐1 macrophages on chamber slides were fixed with 4% paraformaldehyde and permeabilized with 0.1% TRITON X‐100 (Sigma‐Aldrich, catalog no. 93443) each for 10 min. Slides were blocked in 1% BSA/PBS (Fisher Scientific, catalog no. BP9703100) for 30 min before incubating with FLAG M2 antibody (1:50, Millipore, catalog no. F1804) overnight at 4°C. Slides were washed and incubated with secondary goat anti‐mouse IgG1 Alexa Fluor 594 (1:50, Invitrogen, catalog no. A‐21125). Cytoplasmic F‐actin was stained with Phalloidin Alexa Fluor 488 conjugate according to manufacturer instructions (Invitrogen, catalog no. A12379). Nuclear counterstaining was performed with DAPI (ThermoFisher, catalog no. 62248). Images were acquired on the Evident Scientific FV3000 Multi Confocal Microscope system using an inverted Olympus IX83 confocal microscope with a 60× oil PlanApo N SC, 1.40 N.A., 0.15 mm W.D. objective (Evident Scientific MIS). Images were assembled with Fiji (ImageJ). For quantification of cell detachment, images were taken with a 10× objective in the DAPI channel with an EVOS M7000 cell imaging system (ThermoFisher). Forty‐eight images were taken with an automatic image capturing function of an area of 0.5 cm × 1 cm on chamber slides. Analysis was done with the automatic cell count function with Celleste 6 imaging software (ThermoFisher).

### 2.13 Statistical methods

**FIGURE 1:**
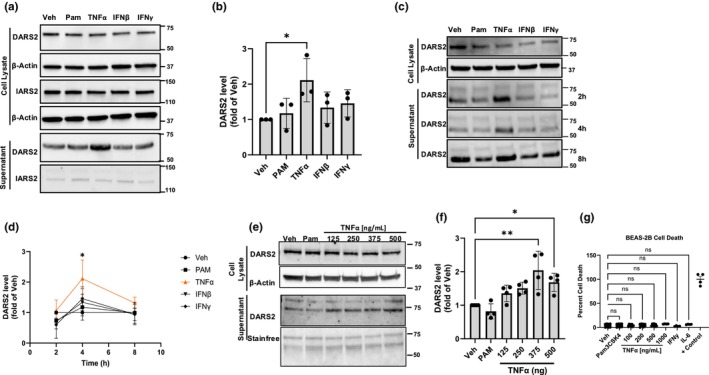
TNFα stimulates DARS2 secretion from airway epithelial cells. (a) Immunoreactive and (b) quantitative levels of DARS2 in the cell lysate (top) and supernatant (bottom) of BEAS2B cells exposed to various stimuli (repeated for *n* = 3 representative blot and quantitation shown). (c) Immunoreactive levels and (d) quantitation of DARS2 in the cell lysate (top) and supernatant (bottom) of primary BEAS‐2B treated with vehicle (Veh), Pam3CSK4 (Pam), TNFα, interferon‐α or interferon‐γ at variable time points (repeated for *n* = 3 representative blot shown data is shown as m ± SD). (e) Levels of DARS2 in the cell lysate (top) and supernatant (bottom) of BEAS‐2B treated with increasing concentrations of TNFα and (f) quantitation (repeated for *n* = 4 representative blot and quantitation shown). (g) Levels of cytotoxicity as measured by LDH release from BEAS2B cells after exposure to various stimuli (*n* = 4 biological replicates/group).

**FIGURE 2:**
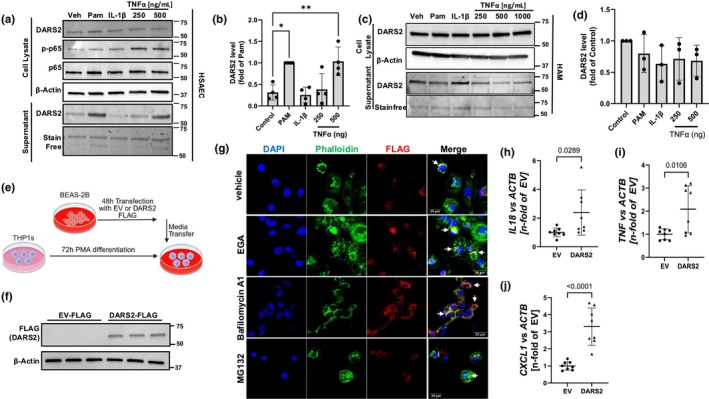
Secreted DARS2 from airway epithelia binds to macrophages. (a) Immunoreactive levels of DARS2 in the cell lysate (top) and supernatant (bottom) and (b) quantitation of DARS2 in the supernatant from primary human small airway epithelial cells (HSAEC) treated with Pam, IL‐1β or increasing concentrations of TNFα (repeated for *n* = 3 representative blots shown). (c) Immunoreactive levels of DARS2 in the cell lysate (top) and supernatant (bottom) and (d) quantitation of DARS2 in the supernatant from human alveolar macrophages (HAM) treated with Pam, IL‐1β or increasing concentrations of TNFα (repeated for *n* = 3 representative blots shown). (e) Schematic of adoptive media transfer from BEAS‐2Bs to recipient PMA‐stimulated THP1‐macrophages. BEAS2Bs were transfected with FLAG‐tagged empty vector (EV) or DARS2‐Flag plasmid and media was transferred to THP‐1 cells. (f) Shown are levels of ectopically expressed DARS2 in THP1 cell lysates (each lane is a biologically independent replicate, repeated for *n* = 2). (g) Immunofluorescence of THP1‐macrophages receiving media from donor BEAS‐2B cells transfected with DARS2‐FLAG as in (e, f) showing co‐localization of DARS2‐Flag with the actin filament marker, phalloidin (arrows, merge panels) Scale bar = 20 μm (repeated for *n* = 2). (h) *IL18*, (i) *TNF*, (j) *CXCL1* mRNA levels by RT‐qPCR analysis in THP1‐macrophages 24 h after receiving media from donor BEAS‐2B cells transfected with EV‐FLAG or DARS2‐FLAG (*n* = 8 biological replicates/group).

**FIGURE 3:**
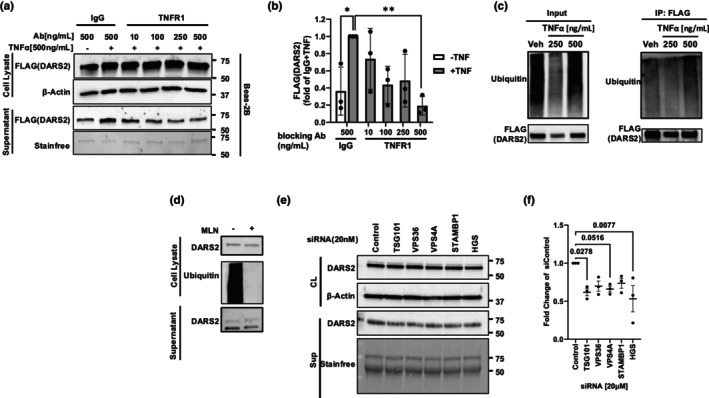
DARS2 secretion is mediated by TNFR1 and ESCRT signaling proteins. (a) DARS2 levels and associated densitometric analysis (b) in the supernatant of BEAS2B cells pretreated with TNFR1 or IgG control blocking Ab and then stimulated with TNFα at various concentrations (repeated for *n* = 3). (c) Ubiquitylation levels of ectopically expressed FLAG‐tagged DARS2 using co‐immunoprecipitation (Co‐IP). Cells were transfected with FLAG‐DARS2 with increasing concentrations of TNFα treatment for 6 h and then processed for FLAG pulldowns followed by probing for ubiquitin. Shown on the left is input and on the right FLAG‐tagged DARS2 ubiquitin levels from Co‐IPs (repeated for *n* = 3). (d) BEAS2B cells were incubated with a pan ubiquitylation inhibitor (MLN); cells and supernatants processed for DARS2 and ubiquitin levels in cell lysates (above) and secreted DARS2 (below) (repeated for *n* = 3). (e) DARS2 protein levels in the cell lysates ([CL], top) and supernatants (bottom) of BEAS2B cells treated with siRNAs against key components of the ESCRT secretion machinery (repeated for *n* = 3). (f) Quantification of supernatant DARS2 (*n* = 3 independent experiments/group).

**FIGURE 4:**
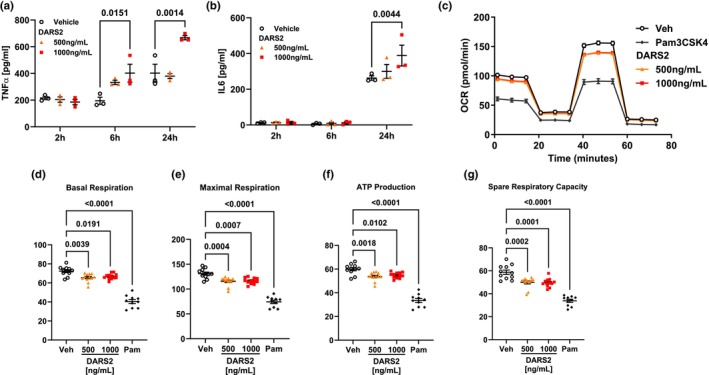
(a) TNFα and (b) IL‐6 concentrations in the supernatant of THP1‐macrophages treated with increasing concentrations of recombinant human DARS2 (*n* = 3 biological replicates per group). (c) Oxygen consumption ratio during the mito‐stress test of THP1‐macrophages treated with increasing concentrations of recombinant human DARS2, Pam, or vehicle (Veh). (d) Basal respiration, (e) maximal respiration, (f) ATP production, and (g) spare respiratory capacity of rhDARS2 treated THP1‐macrophages (*n* = 10–12 biological replicates per group).

For analysis of quantitative data, the following statistical tests were used: a One‐Way ANOVA with Dunnett's multiple comparison was used for data in Figures 1g, 2b, 3d, and 4d–g and Figure S2B. In Figure 2, panels h–j were analyzed using a two‐way Student's *t*‐test. For Figures 3b and 4a,b, a two‐way ANOVA with Tukey's multiple comparison test was used. GraphPad Prism version 10.6.0 was used for statistical analysis.

## 3 RESULTS

### 3.1 Airway epithelial cells secrete DARS2 in response to TNFα

Existing literature demonstrates multiple cell types secrete cytoplasmic tRNA‐synthetases in response to a variety of inflammatory stimuli, including cytokines. To determine if airway epithelial cells release mitochondrial‐derived DARS2 in a comparable manner, we treated BEAS‐2Bs with a panel of cytokines (TNFα, IFNβ, and IFNγ), and immunoreactive DARS2 levels were measured in the supernatant (Figure 1a,b). TNFα induced the most robust secretion into the supernatant from BEAS‐2B cells when measured by immunoblot (Figure 1a bottom). Additionally, BEAS‐2B cells secreted DARS2 into the supernatant in both a time and concentration‐dependent manner in response to TNFα stimulation. Indeed, TNFα stimulation triggered an increase in DARS2 in the supernatant above control levels within 2–4 h of exposure and at high concentrations (Figure 1c–f). To determine if DARS2 release was a function of increased cell death, we measured lactate dehydrogenase concentration in the supernatant and observed no significant effects after TNFα stimulation, indicating that the release of this protein was not secondary to nonspecific cell damage (Figure 1g). Collectively, these data suggest that TNFα robustly induces a regulated release of DARS2 into the supernatant from the airway epithelial cells.

### 3.2 DARS2 secreted from airway epithelial binds to macrophages

We next determined cell type specificity of DARS2 secretion. Thus, human small airway epithelial cells (HSAECs), human bronchial epithelial cells (HBEC), human alveolar macrophages (HAM), and macrophage differentiated THP1‐cells were stimulated with variable concentrations of TNFα (250–1000 ng/mL), and DARS2 levels were measured in the supernatant (Figure 2a–d, Figure S1A,B). DARS2 was secreted into the supernatant maximally after inclusion of TNFα (500 ng/mL) by HSAECs (Figure 2a,b), but not in HAMs, HBECs, or THP1‐cells (Figure 2c,d, Figure S1A,B). In fact, the results suggest that in HAMs and HBECs, TNFα tended to decrease DARS2 release into the supernatant from cells, perhaps suggestive of a feedback inhibition (Figure 2c, Figure S1A). These data indicate DARS2 is secreted selectively from airway epithelia in response to TNFα stimulation. Secreted cytoplasmic tRNA‐synthetases are known to act through paracrine signaling between different cell types. Specifically, Tryptophanyl tRNA‐Synthetase and Lysyl‐tRNA Synthetase secreted from epithelial cells are known to bind to macrophages and dendritic cells (Ahn et al., 2016; Lim et al., 2018). We hypothesized that secreted DARS2 also acts through paracrine signaling once released from the airway epithelia. To test this, we performed adoptive media transfer of supernatants from BEAS‐2B cells to recipient THP1‐differentiated macrophages (Figure 2e). Here, FLAG tagged DARS2, or empty vector (EV) control, was transiently transfected into BEAS‐2B cells; media was changed at 24 h to remove excess plasmid, and at 48 h, cell‐free media was transferred to cultured THP1‐macrophages. Immunoreactive FLAG was detected at 66 kDa (molecular weight of DARS2) in the cell lysate of THP1s receiving media from DARS2‐FLAG transfected BEAS‐2Bs, but no FLAG‐tagged proteins were detected in cells receiving media from EV‐FLAG BEAS‐2B cells (Figure 2f). To confirm uptake of DARS2‐FLAG protein by THP1‐macrophages, supernatant from donor BEAS‐2B cells transfected with DARS2‐FLAG protein was cultured with the addition of an endosomal fusion inhibitor (EGA), a lysosome inhibitor (bafilomycin A1, [BafA1]) or a proteasome inhibitor (MG132) to allow for accumulation of DARS2 following treatment. Cells were then fixed and stained for FLAG and phalloidin (cytoplasmic marker) and counterstained with DAPI (Figure 2g). DARS2 colocalized with the cytoplasmic marker phalloidin, indicating uptake by the THP1‐macrophages. Further, DARS2 accumulated in these cells with the addition of EGA or BafA1 (Figure 2g, merge view, arrows). These data indicate that DARS2 from donor epithelial cells binds to and is internalized by macrophages. This occurs via the endosomal pathway and terminates with lysosomal disposal of DARS2. This accumulation of FLAG was not observed in the THP1‐macrophages receiving conditioned media from EV‐FLAG transfected donor BEAS‐2B cells, indicating that binding and internalization were dependent on DARS2 rather than the FLAG‐tag (Figure S2A). Quantification of DAPI foci per field of view demonstrated that THP‐1 macrophages numbers were not significantly affected by treatment with EGA, Bafilomycin A1, or MG132 (Figure S2B). THP1‐macrophages receiving donor media from DARS2 transfected BEAS‐2B displayed elevated gene expression of *IL18*, *TNF*, and *CXCl1* compared to THP1‐macrophages receiving media from EV‐FLAG transfected BEAS‐2Bs at 24 h post transfer (Figure 2h–j). This suggests DARS2 internalized by the THP1‐macrophages resulting in increased cytokine production and innate immune response in agreement with our previous report (Johnson et al., 2024).

### 3.3 DARS2 secretion is TNFα receptor 1 (TNFR1) and ESCRT pathway dependent

TNFα can signal through either TNFα receptor 1 (TNFR1) or TNFα receptor 2 (TNFR2) (Medler & Wajant, 2019). These receptors have distinct functions depending on cell type and can have opposing effects on inflammation (Papazian et al., 2021; Yang et al., 2019). Soluble TNFα preferentially activates TNFR1 while TNFR2 is primarily activated in response to membrane‐bound TNFα (Medler & Wajant, 2019). Given this, we hypothesized that DARS2 secretion was dependent on TNFR1 signaling. Supernatant was isolated from BEAS‐2Bs after transient transfection with DARS2‐FLAG, incubated with or without TNFα and anti‐TNFR1. Cells exposed to anti‐TNFR1 displayed modestly reduced immunoreactive DARS2‐FLAG compared to those cells incubated with TNFα and an IgG control Ab (Figure 3a,b).

We next investigated the necessity of DARS2 ubiquitylation. Many secreted proteins are marked for intracellular trafficking via ubiquitination. We have previously demonstrated that DARS2 is ubiquitylated through various ubiquitin linkages (Johnson et al., 2024), including those involved in intracellular trafficking. DARS2 was isolated from BEAS‐2Bs via immunoprecipitation after ectopically expressing DARS2‐FLAG, then stimulating cells with increasing concentrations of TNFα. DARS2 immunoprecipitants displayed a concentration‐dependent increase in polyubiquitylation in response to TNFα treatment at 4 and 6 h post treatment (Figure S3A, Figure 3c). In agreement with these data, treatment with a pan‐ubiquitylation inhibitor MLN reduced the baseline secretion of DARS2 into the supernatant (Figure 3d). We previously described DARS2 as being secreted in the exosomal fraction in response to Pam3CSK4 and LPS treatment (Johnson et al., 2024). Likewise, TNFα also induced exosomal release of DARS2 (data not shown). The Endosomal Sorting Complexes Required for Transport (ESCRT) pathway is responsible for the packaging of secreted proteins into multivesicular bodies to be released as exosomes (Dai et al., 2024; Remec Pavlin & Hurley, 2020). Several ESCRT pathway subunits specifically recognize and mediate the release of ubiquitylated cargo. Transient knockdown of ubiquitin‐recognizing several ESCRT proteins tended to reduce DARS2 secretion from cells, but only subunits TSG101 (Strickland et al., 2021) and HGS (Coudert et al., 2021) significantly reduced DARS2 levels in the supernatant of BEAS‐2Bs compared to siControl treated cells (Figure 3e,f). Additionally, knockdown of the ATPase VPS4A, responsible for the scission of cargo from the ESCRT‐III complex during packaging (Remec Pavlin & Hurley, 2020), significantly decreased immunoreactive DARS2 levels in the supernatant of BEAS‐2Bs compared to siControl cells (Figure 3e,f). Knockdown of ESCRT pathway components was confirmed by RT‐qPCR (Figure S3B).

### 3.4 Secreted DARS2 induces M1‐like polarization THP1‐macrophages

Having demonstrated that DARS2 binds to THP1‐macrophages, we next sought to characterize the functional outcomes of secreted DARS2 on these cells. THP1‐macrophages treated with recombinant human DARS2 (rhDARS2), packaged in liposomal vesicles, demonstrated a statistically significant concentration and time‐dependent induction of type‐I cytokines IL‐6 and TNFα into the supernatant (Figure 4a,b). THP1‐macrophages treated with rhDARS2 also demonstrated reduced oxidative phosphorylation compared to vehicle‐treated cells (Figure 4c). Basal respiration, maximal respiration, ATP production, and spare respiratory capacity were all significantly reduced in response to rhDARS2 treatment (Figure 4d–g). M1‐polarized macrophages often display similar metabolic shifts away from oxidative phosphorylation and a preference for glycolysis (Viola et al., 2019; Yu et al., 2023). Overall, these data indicate that rhDARS2 induces THP1‐macrophages towards an M1‐like polarization.

## 4 DISCUSSION

Regulated secretion of tRNA‐synthetases to fine‐tune the immune response is well described, but the mechanism of release for many of these synthetases remains to be identified. The new contributions of these studies show that (i) DARS2 is secreted from epithelial cells in response to TNFα and binds to macrophages to induce further cytokine secretion in a positive feedback loop and (ii) DARS2 is released in a ubiquitination and ESCRT pathway‐dependent manner. Through these pathways, we expand our understanding of DARS2's role as a secreted immunostimulatory protein.

Here we demonstrate a novel paracrine feedback control loop driven by DARS2 secretion. DARS2 is released from lung epithelial cells after TNFα, binds, and is internalized within macrophages, and appears to induce an M1‐like polarization, further stimulating TNFα release (Figure 4). Positive and negative feedback loops are essential mechanisms of cell homeostasis, and cross talk between epithelia and macrophages is well described. Many components of the immune response within host defense are coordinated by complex networks of positive and negative feedback loops that fine‐tune the host immune response. Several cytoplasmic tRNA‐synthetases function in this manner. For example, Glutamyl‐Prolyl‐tRNA Synthetase (EPRS) is known to act as a negative regulator of inflammation. In response to interferon gamma (IFNγ) stimulation, EPRS dissociates from the multi‐synthetase complex and associates with the IFN‐γ‐activated inhibitor of translation (GAIT) complex, dampening further IFNγ signaling through translation repression of IFN‐induced genes (Arif et al., 2009, 2018). Conversely, KARS is secreted from multiple cell types in response to TNFα and, in a feed‐forward mechanism, triggers TNFα secretion from macrophages (Park et al., 2005). However, a unique aspect of our studies is that DARS2 release in response to TNFα stimulation and its ability to potentiate this cytokine release represents the first mitochondrial tRNA‐synthetase described to act in this manner. This novel role for DARS2 demonstrates its importance in fine‐tuning the immune response by the mitochondrial apparatus linking tRNA‐synthetases to cytokine signaling. Further areas for investigation remain, such as how DARS2 secretion is downregulated through negative feedback control mechanisms that temper the immune response, perhaps by known pro‐resolving mediators (e.g., TGFβ and IL‐10). We speculate that there may be inhibitory factors that can be discovered through proteomic approaches that, via specific molecular cues, facilitate inhibition of DARS2 release from epithelia.

These results also are the first to identify the secretion of DARS2, or other tRNA synthetases, as dependent on the ESCRT pathway. We show that DARS2 is ubiquitylated in response to TNFα and its secretion is partially dependent on this modification. Likewise, DARS2 secretion was significantly impaired in response to knockdown of ESCRT components which recognize ubiquitylated cargo. Interestingly, knockdown of the ESCRT protein ATPase VPS4A significantly impaired DARS2 secretion. These data indicate that disruption of the substrate recognition or terminal packaging steps of the ESCRT pathways significantly impairs DARS2 release. Further studies should seek to identify the motifs in DARS2 required for its recognition and packaging by the ESCRT machinery. The generalizability of the ESCRT pathway as a mechanism of tRNA synthetase secretion is also of interest. Many secreted tRNA synthetase display an affinity for signaling through TLR2 and TLR4 dependent pathways (Fernandez et al., 2013; Jin, 2019; Qi et al., 2024) indicating a level of similarity in their molecular behavior. Given the highly conserved nature of many tRNA synthetase proteins it is not unreasonable to hypothesize that the ESCRT components may recognize other tRNA synthetases to facilitate their export.

## 5 CONCLUSION

In summary, these data suggest that we have uncovered a previously unrecognized ability of a mitochondrial synthase to be released from human airway epithelia that in turn acts in a paracrine mechanism to control some aspects of the innate immune pathway and cellular energetics with myeloid‐derived cells.

## FUNDING INFORMATION

This facility is supported in part by grant P30 CA016058, National Cancer Institute, Bethesda, MD. This work was supported by P01HL114453, R01HL097376, R01HL081784, and R01HL096376 awarded to RKM.

## CONFLICT OF INTEREST STATEMENT

The authors have declared that no conflict of interest exists.

## ETHICS STATEMENT

No human or animal subjects were involved in this study.

## Supporting information


Figure S1.

Figure S2.

Figure S3.

Table S1.

Table S2.

## Data Availability

Authors will provide all raw data associated with this manuscript upon request. Data is available in Excel, and all full unaltered immunoblots are available upon request.
