# Supplementary material for: Secreted mitochondrial aspartyl‐tRNA synthetase (DARS2) regulates TNFα signaling
Source: Physiol Rep. 2025 Nov 10;13(21):e70627. doi: 10.14814/phy2.70627 (PMC12602254; doi:10.14814/phy2.70627)
Supplement: Supplementary file 1 — Figure S1. [file PHY2-13-e70627-s002.pdf]

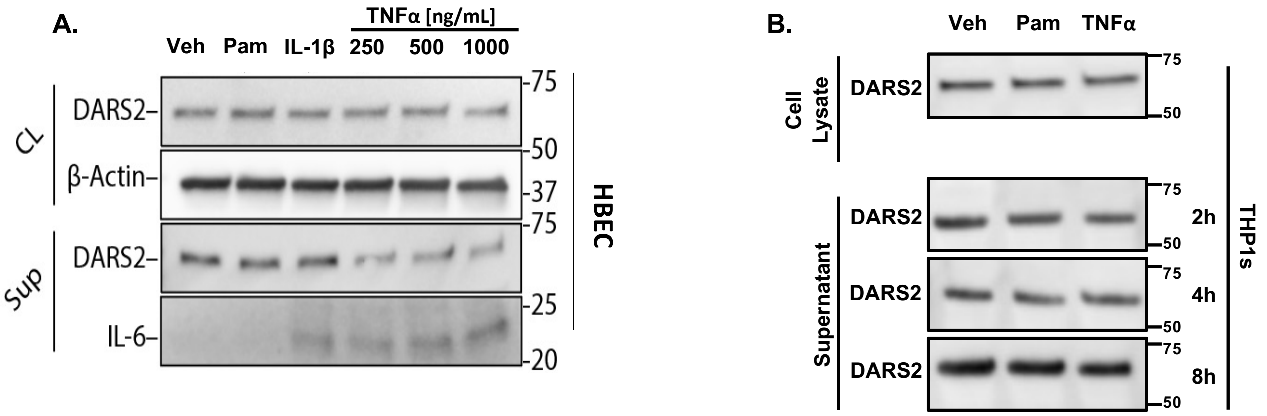

Figure S1. (A) Immunoreactive levels of DARS2 in the cell lysate (top) and supernatant (bottom) of primary human bronchial epithelial cells (HBEC)(*n* = 3). **(B)** Immunoreactive levels of DARS2 in the cell lysate (top) and supernatant (bottom) of THP1-macrophages treated with vehicle (Veh), Pam3CSK4 (Pam) or TNFα at variable time points (*n* = 2).
