# Supplementary material for: Secreted mitochondrial aspartyl‐tRNA synthetase (DARS2) regulates TNFα signaling
Source: Physiol Rep. 2025 Nov 10;13(21):e70627. doi: 10.14814/phy2.70627 (PMC12602254; doi:10.14814/phy2.70627)
Supplement: Supplementary file 2 — Figure S2. [file PHY2-13-e70627-s005.pdf]

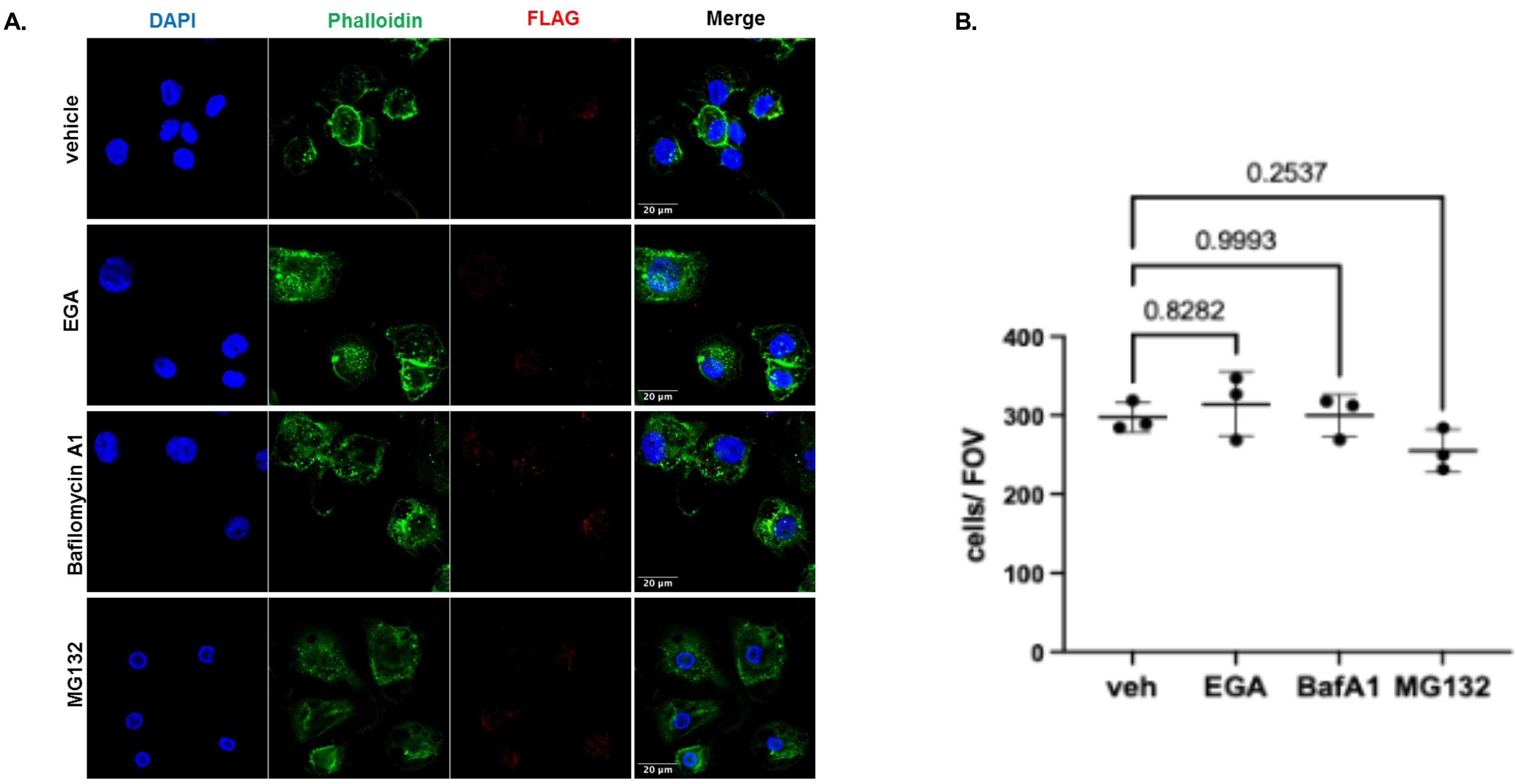

Figure S2. (A) Immunofluorescence of THP1-macrophages receiving media from donor BEAS-2B cells transfected with DARS2-FLAG as in (**Fig. 2C-E**) showing lack of co-localization of EV-Flag with the actin filament marker, phalloidin (Scale bar=20 µm (*n*=2). **(B)** Quantification of number of cells per field of view following treatment with chemical inhibitors to assess cell detachment.
