# Supplementary material for: Secreted mitochondrial aspartyl‐tRNA synthetase (DARS2) regulates TNFα signaling
Source: Physiol Rep. 2025 Nov 10;13(21):e70627. doi: 10.14814/phy2.70627 (PMC12602254; doi:10.14814/phy2.70627)
Supplement: Supplementary file 3 — Figure S3. [file PHY2-13-e70627-s004.pdf]

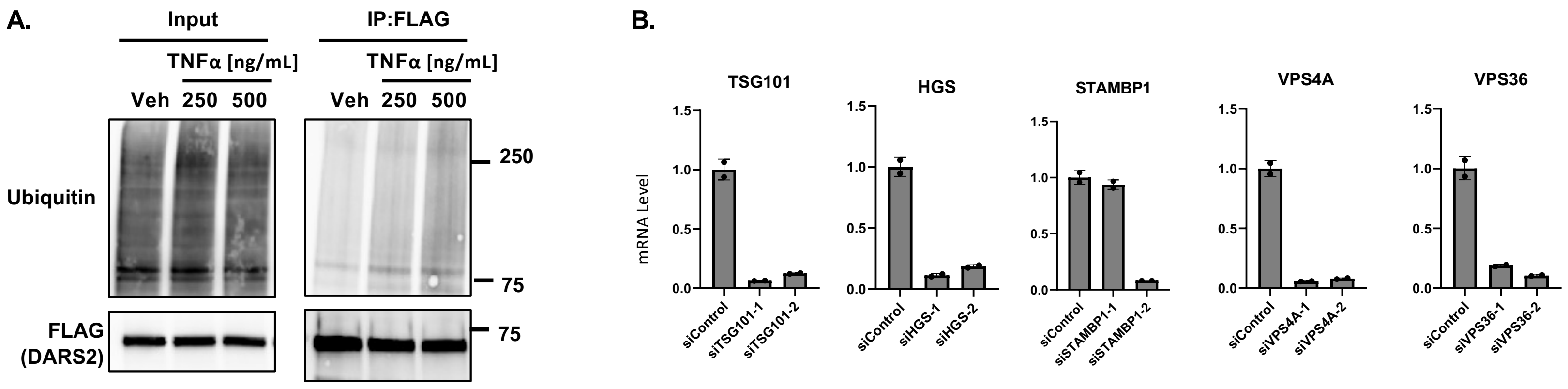

Figure S3. (A) Ubiquitylation levels of ectopically expressed FLAG-tagged-DARS2 using immunoprecipitation following TNFα treatment for 4h. Cells were transfected with FLAG-DARS2 with increasing concentrations of TNFα and then processed for FLAG pulldowns followed by probing for ubiquitin. Shown on the left is input and on the right ubiquitin levels from Co-IPs. (*n*=3). **(B)** *TSG101, HGS, STAMBP1, VPS4A* and *VPS36* mRNA levels by RT-qPCR analysis in BEAS2B cells following treatment of siRNA against that target compared to control siRNA (*n*=2 biological replicates).
