# Supplementary material for: Secreted mitochondrial aspartyl‐tRNA synthetase (DARS2) regulates TNFα signaling
Source: Physiol Rep. 2025 Nov 10;13(21):e70627. doi: 10.14814/phy2.70627 (PMC12602254; doi:10.14814/phy2.70627)
Supplement: Supplementary file 4 — Table S1. [file PHY2-13-e70627-s003.docx]

**Supplemental Table 1**: **RT-qPCR Primers Used in the Study**

| **gene** | **template strand** | **sequence** |
| --- | --- | --- |
| TSG101 | Forward | CGGGGCCACCAAATACTTCC |
|  | Reverse | CACCAGGTGGGTAAGGACAG |
| VPS36 | Forward | ACGATGGCGAGGAGAAGATAAA |
|  | Reverse | AGAATGGCCATGCAACACTC |
| STAMBP1 | Forward | TGGGCTGGATTCATACTCACC |
|  | Reverse | CTTGGGGGAGCAAACAATGG |
| VPS4A | Forward | GAGAACCAGAGTGAGGGCAAG |
|  | Reverse | CCATCACGACGGCACCCAT |
| HGS | Forward | ACGAGCCCAAGTACAAGGTG |
|  | Reverse | AGTCTGGGGCTCTCTCGG |
| TNF | Forward | CTGCACTTTGGAGTGATCG |
|  | Reverse | AAGATGATCTGACTGCCTGG |
| CXCL1 | Forward | CCCAAGAACATCCAAAGTGTG |
|  | Reverse | CATTCTTGAGTGTGGCATGAC |
| IL18 | Forward | TGACCAAGTTCTCTTCATTGAC |
|  | Reverse | GGTGCATTATCTCTACAGTCAG |
| ACTB | Forward | CACCATTGGCAATGAGCGGTTC |
|  | Reverse | AGGTCTTTGCGGATGTCCACGT |
