# Supplementary material for: Secreted mitochondrial aspartyl‐tRNA synthetase (DARS2) regulates TNFα signaling
Source: Physiol Rep. 2025 Nov 10;13(21):e70627. doi: 10.14814/phy2.70627 (PMC12602254; doi:10.14814/phy2.70627)
Supplement: Supplementary file 5 — Table S2. [file PHY2-13-e70627-s001.docx]

**Supplemental Table 2: RNAi constructs used in this study**

| **RNAi constructs** | **Sequence** |
| --- | --- |
| hs.Ri.TSG101.13.1-SEQ1 | rCrArGrUrCrUrGrArArArArCrArArUrGrArUrArUrCrGrATG |
| hs.Ri.TSG101.13.1-SEQ2 | rCrArUrCrGrArUrArUrCrArUrUrGrUrUrUrUrCrArGrArCrUrGrArU |
| hs.Ri.TSG101.13.3-SEQ1 | rGrUrUrUrArUrCrArUrUrCrArArGrUrGrUrArArArArUrATT |
| hs.Ri.TSG101.13.3-SEQ2 | rArArUrArUrUrUrUrArCrArCrUrUrGrArArUrGrArUrArArArCrUrG |
| hs.Ri.STAMBP.13.1-SEQ1 | rGrCrArUrUrGrGrUrCrUrUrUrGrArUrArCrCrUrArArGrAAT |
| hs.Ri.STAMBP.13.1-SEQ2 | rArUrUrCrUrUrArGrGrUrArUrCrArArArGrArCrCrArArUrGrCrArG |
| hs.Ri.STAMBP.13.2-SEQ1 | rArCrGrCrUrCrUrUrUrArUrUrGrArGrArArArCrUrArCrCAA |
| hs.Ri.STAMBP.13.2-SEQ2 | rUrUrGrGrUrArGrUrUrUrCrUrCrArArUrArArArGrArGrCrGrUrGrA |
| hs.Ri.VPS4A.13.2-SEQ1 | rGrArArGrGrArUrUrArUrUrUrArCrGrArArGrCrArArArGAG |
| hs.Ri.VPS4A.13.2-SEQ2 | rCrUrCrUrUrUrGrCrUrUrCrGrUrArArArUrArArUrCrCrUrUrCrArG |
| hs.Ri.VPS4A.13.3-SEQ1 | rCrCrArArUrCrArArArUrUrCrCrCrArCrArCrUrUrGrUrUCA |
| hs.Ri.VPS4A.13.3-SEQ2 | rUrGrArArCrArArGrUrGrUrGrGrGrArArUrUrUrGrArUrUrGrGrCrA |
| hs.Ri.VPS36.13.1-SEQ1 | rGrUrCrArUrGrGrUrArArUrUrGrArGrCrUrUrCrArGrUrCTC |
| hs.Ri.VPS36.13.1-SEQ2 | rGrArGrArCrUrGrArArGrCrUrCrArArUrUrArCrCrArUrGrArCrGrC |
| hs.Ri.VPS36.13.3-SEQ1 | rArArGrArUrGrArGrArCrCrArUrCrArGrGrUrUrUrArArATC |
| hs.Ri.VPS36.13.3-SEQ2 | rGrArUrUrUrArArArCrCrUrGrArUrGrGrUrCrUrCrArUrCrUrUrCrU |
| hs.Ri.HGS.13.3-SEQ1 | rCrUrArCrCrArGrCrCrUrUrArCrArArCrArUrGrCrArGrAAT |
| hs.Ri.HGS.13.3-SEQ2 | rArUrUrCrUrGrCrArUrGrUrUrGrUrArArGrGrCrUrGrGrUrArGrCrC |
| hs.Ri.HGS.13.4-SEQ1 | rArArGrArGrArCrArArGrUrGrGrArGrGrUrArArArCrGrUCC |
| hs.Ri.HGS.13.4-SEQ2 | rGrGrArCrGrUrUrUrArCrCrUrCrCrArCrUrUrGrUrCrUrCrUrUrCrA |
